# The Intervention Effect of Traditional Chinese Medicine on the Intestinal Flora and Its Metabolites in Glycolipid Metabolic Disorders

**DOI:** 10.1155/2019/2958920

**Published:** 2019-06-04

**Authors:** Sha Di, Yitian Wang, Lin Han, Qi Bao, Zezheng Gao, Qing Wang, Yingying Yang, Linhua Zhao, Xiaolin Tong

**Affiliations:** ^1^Department of Endocrinology, Guang'anmen Hospital, China Academy of Chinese Medical Sciences, Beijing 100054, China; ^2^Shenzhen Hospital, Guangzhou University of Chinese Medicine, Guangzhou 518034, China

## Abstract

Metabolic syndrome (MS), which includes metabolic disorders such as protein disorder, glucose disorder, lipid disorder, and carbohydrate disorder, has been growing rapidly around the world. Glycolipid disorders are a main type of metabolic syndrome and are characterized by abdominal obesity and abnormal metabolic disorders of lipid, glucose, and carbohydrate utilization, which can cause cardiovascular and cerebrovascular diseases. Glycolipid disorders are closely related to intestinal flora and its metabolites. However, studies about the biological mechanisms of the intestinal flora and its metabolites with glycolipid disorders have not been clear. When glycolipid disorders are treated with drugs, a challenging problem is side effects. Traditional Chinese medicine (TCM) and dietary supplements have fewer side effects to treat it. Numerous basic and clinical studies have confirmed that TCM decoctions, Chinese medicine monomers, or compounds can treat glycolipid disorders and reduce the incidence of cardiovascular disease. In this study, we reviewed the relationship between the intestinal flora and its metabolites in glycolipid metabolic disorders and the effect of TCM in treating glycolipid metabolic disorders through the intestinal flora and its metabolites. This review provides new perspectives and strategies for future glycolipid disorders research and treatment.

## 1. Introduction

Metabolic syndrome (MS) comprises a cluster of metabolic disorders such as protein disorder, glucose disorder, lipid disorder, and carbohydrate disorder. Glycolipid metabolic disorders are the main type of MS and are characterized by abdominal obesity and abnormal metabolic disorders of lipids and carbohydrates in blood, including T2DM, obesity, hyperlipidemia, and insulin resistance. Glycolipid metabolic disorders are more complicated, because of multifactorial interaction, than single factor metabolic abnormalities and are the primary risk factors for cardiovascular and cerebrovascular diseases [1]. Therefore, a single intervention such as only reducing hyperglycemia or hypertension cannot effectively regulate multiple metabolic disorders.* China Experts on Type 2 Diabetes with Dyslipidemia*, published in 2017, reported that 42% T2DM patients have dyslipidemia, and the overall compliance rate is only 12% among patients treated with drugs [2]. Exploring an integrated prevention strategy has always been a problem for the medical community.

The intestinal flora consists of approximately 1014 bacteria that exist primarily in the large intestine. Intestinal flora has an important role in a host's physiology such as modulation of immunity development, inflammation, and energy metabolism. Intestinal flora's imbalance can contribute to diseases development, including obesity, hyperlipemia, T2DM, cardiovascular disease, irritable bowel syndrome, liver diseases, and skin diseases [3]. The resident microbiota produces a multitude of metabolites, which are employed to regulate cellular function [4]. Intestinal flora with its metabolites may provide us a new sight to explore the possible mechanisms of diseases. It may also identify many potential diagnostic and therapeutic targets. Ze-Hua Zhao reported that intestinal flora's metabolites, as mediators in the gut-liver axis, reveal the vital role of host-diet-microbiota-metabolite interactions in nonalcoholic fatty liver disease (NAFLA) [5]. The association of intestinal flora's metabolites from carbohydrate fermentation and protein fermentation in NAFLA, insulin resistance, and T2DM, has been reviewed by Emanuel E. Canfora [6].

16S ribosomal RNA (16S rRNA) gene sequencing and metagenomic sequencing methods have been employed in quantitative analysis of intestinal flora since the 1990s. The methods provide an effective approach to study intestinal flora's variation and function. 16S rRNA uses probes or primers designed to detect intestinal flora to specify which phylum, group, genus, or species it belonging to. Metagenomic sequencing uses taxonomically informative gene tags to target and amplify genomes to study intestinal flora's composite and function [7]. Metabolomics studies have been utilized to seek biomarkers and elucidate the pathophysiology and progression of diseases, such as insulin resistance [8], T2DM [9], and NAFLD [10], and predict pharmacokinetic profiles in vivo and evaluate the effectiveness and safety of drugs [11]. Connection of genomics and metabolomics provides us with more understanding of the relationship between gut microbiota and its metabolites and has also been applied in disease diagnostics and prognosis in precision medicine [12].

Traditional Chinese medicine (TCM) is an effective method for treating MS. Many basic and clinical studies have confirmed that Chinese decoctions, Chinese medicine monomers, or compounds can reduce lipid and carbohydrate in blood and decrease the incidence of cardiovascular disease. For people with abnormal glycolipid metabolic disorders, TCM has the clinical efficacy advantage of “multitarget treatment.” Recent studies have demonstrated that TCM regulated lipid and carbohydrate by adjusting structure of disordered intestinal flora. For example, GeGenQilian decoction, Jiangtangtiaozhi formula, and other designed herbal formulas lowered carbohydrate level and regulated intestinal flora [13, 14]. However, most current studies are limited to analyze the intestinal genomics and detect clinical indicators and do not explore the potential relationship between the effects of TCM on the intestinal flora and metabolites [15–17]. A large gap remains regarding the exploration of changes in the biological mechanisms of the flora and its metabolites. This review describes the relationship of the intestinal flora and its metabolites in glycolipid metabolic disorders, and the mechanisms of TCM in treating glycolipid metabolic disorders through the intestinal flora and its metabolites.

## 2. The Relationship of Intestinal Flora and Its Metabolites in Glycolipid Metabolism

The intestinal flora has an important role in the pathophysiology of glycolipid metabolic diseases such as obesity and T2DM [18–20]. Patients with abnormal glycolipid metabolism have decreased intestinal bacterial diversity and gene richness. Transplantation of intestinal flora from obese patients can cause obesity in sterile mice, which suggests that the intestinal flora disorder can cause obesity and metabolic diseases [19]. In addition, the inoculation of the* Enterobacter cloacae B29 strain*, which was isolated from the stool of severe obese patients, into sterile mice can cause severe obesity and insulin resistance [21]. Compared with healthy people, patients with T2DM had a moderate imbalance in the intestinal flora [22, 23]. An analysis of the gut flora of 292 Danish individuals also showed that individuals with low microbial abundance often had more proinflammatory bacteria, and most of them had insulin resistance, high triglycerides level, and had a higher risk of T2DM [24]. To date, studies have demonstrated that the structural imbalance in the intestinal flora primarily affects the metabolism of glycolipid in the body, which includes the metabolism of short-chain fatty acids (SCFAs), bile acids (BAs), choline, amino acids, and other metabolites.

### 2.1. Fatty Acids Metabolism and the Intestinal Flora

Fatty acids associated with energy metabolism, lipid metabolism, and cell membrane's constituents also act as signal molecules. Abnormal fatty acids metabolism leads to metabolic diseases such as T2DM, nonalcoholic hepatic steatosis, hyperlipemia, and obesity.

#### 2.1.1. SCFAs Metabolism and the Intestinal Flora

SCFAs absorbed in the colon are major metabolites produced by anaerobic gut microbiota through the fermentation of indigestible carbohydrates such as polysaccharides, oligosaccharides, and fiber [25]. The SCFAs are produced in the colonic lumen and are transported across the epithelium by diffusion. When transported into colonic tissue, they are primarily converted by the colonic epithelium into lipids or ketone bodies such as *β*-hydroxybutyrate or acetoacetate [26].

SCFAs, which primarily consist of acetate, propionate, and butyrate (in a ratio of approximately 60:20:20 in the colon) [27], are thought to play a vital role in metabolism, inflammation, immune homeostasis, neural pathways, and cancer [28–31]. As for glycolipid metabolism, SCFAs can provide energy for various human tissues to maintain intestinal epithelial cell function, glucose homeostasis, and insulin sensitivity, which affects glucose, lipid, and cholesterol metabolism. At the same time, SCFAs used as substrates for gluconeogenesis also contribute to T2DM, obesity, hyperlipidemia, and other metabolic diseases by increasing a host's energy harvest [32].

SCFAs regulate glycolipid metabolism and are substrates that bind to G protein-coupled receptors (GPRs), including GPR41 (i.e., free fatty acid receptor 3 [FFAR3]), GPR43 (i.e., free fatty acid receptor 2 [FFAR2]), and GPR109A (i.e., hydroxycarboxylic acid receptor 2 [HCA2]), which are associated with inflammation, immunity, and cancer. Gut microbiota promotes adiposity and body weight via GPR41. However, SCFAs may prevent obesity by activating GPR43 [33]. GPR43 is associated with metabolic activity of the gut microbiota with host body energy homoeostasis processes, such as glycolipid metabolism, via suppressing insulin signaling in adipocytes [34].* Firmicutes*,* Bacteroidetes*, and* Actinobacteria *are the primary phyla and* Butyricicoccus*,* Blautia*, and* Phascolarctobacterium* are the primary genera in the gut microbiota that have an important role in SCFAs metabolism [35, 36]. In addition, SCFAs can be produced by bacteria in the genera* Bacteroides, Bifidobacterium, Propionibacterium, Eubacterium*,* Lactobacillus, Clostridium, Roseburia, Lachnospira, an*d* Prevotella *[37, 38]. SCFAs are positively associated with* Prevotella*,* Alistipes*, and* Barnesiella* and negatively associated with* Bacteroides* and* Enterococcus*.* Prevotellaceae* bacteria are enriched in obesity [39].* Lachnospira,* as SCFA producer, has a negative correlation with body weight [37]. Fecal SCFA level showed negative relation with bacterial diversity, and 70 unique microbial taxa had relationship with at least one SCFAs (acetate, butyrate, or propionate). Jacobo de la Cuesta-Zuluaga studied the relationship of fecal SCFAs, gut microbiota, gut permeability, and cardiometabolic outcomes, including obesity and hypertension in 441 community-dwelling adults. High fecal SCFAs, obesity, and cardiometabolic disease risk factors represented higher abundance of several disease-associated microbiota, including* Enterobacter hormaechei*,* Haemophilus parainfluenzae*,* Streptococcus, *and* SMB53*. Lower fecal SCFAs showed higher abundance of some beneficial microbes.* Christensenellaceae, Methanobrevibacter,* and* Oscillospira *had relationship with lower BMI, weight.* Alistipes* and* Bacteroides* associated with decreased obesity [40].

Acetate and propionate are primarily produced by the* Bacteroidetes* phylum bacteria, and butyrate is primarily produced by the* Firmicutes* phylum bacteria [41]. However, the gut microbiota has a complicated relationship with the SCFAs. For example, Murphy et al. showed that* Firmicutes, Bacteroidetes*, and* Actinobacteria* phyla bacteria were not associated with energy harvest and that the fecal SCFAs levels and fecal energy content in high-fat diet- (HFD-) fed mice were also not associated [42]. Acetate, the predominant SCFAs, is produced by anaerobic intestinal microbiota such as the* Firmicutes *phylum,* Bacteroides* (e.g.,* Bacteroides thetaiotaomicron*),* Proteobacteria* (e.g.,* Desulfovibrio piger*),* Bifidobacterium*, and* Clostridia* [43]. Acetate can strengthen tight junctions of epithelial cells with mediation a* Bifidobacterium*-induced improvement in the intestinal barrier against bacterial endotoxin [44]. Acetate provides energy for a host and is adsorbed by tissues involved in the synthesis of cholesterol, long-chain fatty acids, glutamine, and glutamate [45]. Acetate is positively associated with the phylum* Tenericutes* and the families* Christensenellaceae*, unclassified* Clostridiales, Peptococcaceae*, and* Clostridiaceae*. And, it is negatively associated with the genera* Blauti*a* Oscillospira* [46], unclassified family in* Clostridiales, Defluviitaleaceae,* and* Porphyromonadaceae* in HFD-induced obesity [47].

Propionate and butyrate lower than acetate inhibit food intake [48]. Propionate and butyrate formation is associated with* Lachnospiraceae*,* Ruminococcaceae*, and* Negativicutes* (all members of the phylum* Firmicutes*) and the phyla* Verrucomicrobia* and* Bacteroidetes* [49]. The abundance of* Bacteroidetes* is positively associated with propionate and butyrate. Butyrate provides energy for colonic epithelial cells and suppresses inflammation, which is primarily caused by lipopolysaccharides [50]. 50% propionate, as a precursor for gluconeogenesis, is used as the substrate for hepatic gluconeogenesis [51]. Propionate produced by gut microbiota prevented adipose tissue accumulation in lean twin transplanted mice [52]. Propionate and butyrate decrease the incidence of diet-induced obesity by regulating gut hormone release [48].

Butyrate produced by gut microbiota primarily provides energy for colonic cells [53] and is primarily produced by* Clostridium clusters IV* and* XIVa *species of the genera* Eubacterium* and* Butyrivibrio *in the phylum* Firmicutes* [26]. Butyrate enters the mitochondria of colonocytes and undergoes *β*-oxidation to acetyl-CoA, which participates in the tricarboxylic acid (TCA) cycle. The level of butyrate decreased in HFD-induced rats [54]. Propionate and butyrate stimulate the release of peptide YY (PYY) and the hormones glucagon-like peptide 1 (GLP-1) from intestinal L-cells, and thereby regulate food intake and glucose tolerance [55]. PYY produced by GPR41 is an enteroendocrine cell-derived hormone such as the incretins GLP-1 and glucose-dependent insulinotropic polypeptide, which normally inhibits gut motility and thereby absorbs more energy from feces, decreases the intestinal transit rate, and increases the harvest of energy from the diet [56]. GPR41 plays a vital role in the butyrate stimulation of GLP-1. GPR41-deficient mice have a normal body weight and glucose homeostasis [48]. Increased butyrate excretion was in positively correlation with the abundances of* Faecalibacterium prausnitzii*,* Roseburia faecis*, and other* Clostridiales, *known as butyrate-producers, as well as* Enterobacter hormaechei*,* Haemophilus parainfluenzae*, and* Streptococcus*, winch were unknown for fermentation capacity in obesity and hypertension of 441 community-dwelling adults. Almost microbiota significantly associated with butyrate excretion was also connected with acetate or propionate excretion [40].

In addition, SCFAs stimulate GLP-2 in the ileum, which decreases gut barrier permeability and lipopolysaccharide level. Propionate is primarily delivered and used by the liver, which has an important role in gluconeogenesis. Butyrate, as substrate, binds to GPR109a and is associated with the gut microbiota. Gpr109a is not expressed in the colons of germ-free and antibiotic-treated mice. The drug tributyrin, which can be metabolized into butyrate, induces GPR109a expression in antibiotic-treated animals [26].

#### 2.1.2. The Metabolism of Other Fatty Acids and the Intestinal Flora

Unsaturated fatty acids can inhibit lipotoxicity caused by palmitic acid (16:0) [57]. Saturated fatty acids are risk factors for developing obesity and its associated disorders. Polyunsaturated fatty acids and monounsaturated fatty acids can inhibit the hepatic steatosis process [58]. Free fatty acids (FFAs) are associated with obesity, inflammation, altered glucose homeostasis, and cardiovascular disease. The genus* Akkermansia* is negatively associated with FFAs, and an alteration in FFAs levels is associated with imbalanced levels of* Akkermansia* and* Lactobacillus* [59]. Patients with MS have higher levels of saturated fatty acids, FFAs, monounsaturated fat, and trans fat (i.e., trans-fatty acids) [60, 61].

Gut microbiota lowers fatty acids oxidation by inhibiting the production of adenosine monophosphate kinase, which has a vital role in energy metabolism. Increased fatty acids oxidation enhances the cellular energy status, and increased insulin sensitivity decreases the glycogen level. Germ-free mice have increased adenosine monophosphate kinase levels in the liver and skeletal tissues, and enhanced fatty acids oxidation [62]. Monounsaturated fatty acids and saturated fatty acids in fasting serum are associated with the increased abundance of* Blauti*a and* Dorea* bacteria, and decreased levels of* Coprococcus* and* Peptococcaceae* bacteria [46, 61].

### 2.2. Bile Acids Metabolism and the Intestinal Flora

Primary BAs are the main ingredient of bile and are synthesized from cholesterol in the liver through two major bile acid synthetic pathways: the classic pathway and the alternative pathway [63]. These acids are collected in the gall bladder and further released into the duodenum after a meal through bile. This action promotes the metabolism of dietary lipids and lipid-soluble vitamins. The duodenum, jejunum, and proximal ileum have high concentrations of bile salts. The proximal ileum digests and absorbs fat, and the distal ileum absorbs bile salts. Small bowel microbes promote bile salt metabolism via deconjugation and hydroxy group oxidation. More than 95% bile salts are absorbed by the ileum and cycled into the liver—a process called enterohepatic circulation. The remaining 5% of BAs escape the enterohepatic circulation are converted into secondary BAs, which include mostly deoxycholic acid (i.e., 3*α*,12*α*-dihydroxy-5*β*-cholan-24-oic acid) and lithocholic acid (i.e., 3*α*-hydroxy-5*β*-cholan-24-oic acid). These acids enhance toxicity and are more easily absorbed by the intestine and gut microbiota, especially the* Firmicutes* phyla, with bacterial enzymes in the large intestine [32, 64].

The BAs, as signaling molecules, regulate BAs metabolism, lipid utilization, glucose utilization, energy, drug metabolism, inflammation, detoxification, and immunity by activating nuclear receptors. These nuclear receptors include the Farnesoid X receptor (FXR), cell surface GPRs, constitutive androstane receptor, vitamin D receptor, and pregnane X receptor. Cell surface GPRs also include the G protein-coupled bile acids receptors (e.g., TGR5 and GPBAR-1) [63–65]. FXR, an intracellular BAs sensor and transcriptional regulator, regulates BAs homeostasis and lipid homeostasis. Serum BAs, cholesterol, triglycerides, hepatic cholesterol, triglycerides, and a proatherogenic serum lipoprotein profile are dramatically increased in FXR/BAR-null mice than in wild-type mice [66]. Intestine-specific activation of FXR can alleviate obesity and metabolic deterioration phenotype in mice and can promote body heat production and white fat browning [67]. TGR5 activated by BAs increases energy expenditure and improves obesity caused by diet. In addition, TGR5 signaling promotes GLP-1 release, which improves liver and pancreatic function and glucose tolerance in obese mice [68].

Gut microbiota regulate the biotransformation of BAs by deconjugation, dehydroxylation, and reconjugation, which change BAs composition and modulate FXR and TGR5 signaling. There are four gut microbiota phyla in the small intestine, including* Clostridium*,* Bacteroides*,* Lactobacillus*,* Bifidobacterium*, and* Enterococcus*, which secrete bacterial bile salt hydrolase enzyme that deconjugates primary BAs in the small intestine [69]. Some gut microbiota is involved in BAs metabolism. For example,* Bacteroides*,* Bifidobacterium*,* Clostridium*,* Lactobacillus*, and* Listeria* are involved in deconjugation.* Bacteroides*,* Clostridium*,* Escherichia*,* Eggerthella*,* Eubacterium*,* Peptostreptococcus*, and* Ruminococcus* are involved in oxidation and epimerization.* Clostridium*, and* Eubacterium* are involved in 7-dehydroxylation.* Bacteroides*,* Eubacterium*, and* Lactobacillus* are involved in esterification. And* Clostridium*,* Fusobacterium*,* Peptococcus*, and* Pseudomonas* are involved in desulfation [70].* Lactobacillus* can produce bile salt hydroxylase [71]. Fecal secondary BAs are associated with* Clostridium* cluster XVIa bacteria [72].

Gut microbiota play a role in transforming primary BAs, which is involved in lipid and glucose metabolism [73]. Mice treated with tempol, which can prevent obesity, had increased the level of Bacteroidetes bacteria and decreased the level of* Firmicutes* and* Lactobacillus* bacteria, and synthesized more BAs that regulated the FXR [71]. Rats injected with irinotecan (i.e., CPT-11) have disturbance in the intestinal microbiota, which results in quantitative and qualitative changes in the BAs. Serum 12*α*-hydroxylated BAs and their conjugates increased and were correlated with insulin resistance in diabetic patients [74]. Cholic acid (i.e., 3*α*, 7*α*, 12*α*-trihydroxy-5*β*-cholan-24-oic acid) and chenodeoxycholic acid (i.e., 3*α*, 7*α*-dihydroxy-5*β*-cholan-24-oic acid) are primary BAs and synthesized in the liver. They conjugate with glycine or taurine and convert the molecules into conjugated BAs or bile salts. The levels of deoxycholic acid and taurodeoxycholic acid were significantly increased in plasma and liver tissues, and deoxycholic acid also raised in intestinal tissues and feces in obesity rat, which were connection with enhancement of genera* Blautia, Coprococcus, Intestinimonas, Lactococcus, Roseburia,* and* Ruminococcus* [75, 76].

### 2.3. Choline Metabolism and the Intestinal Flora

Choline is a water-soluble nutrient that is essential for human life [77]. It is also a vital ingredient of cell membranes, mitochondrial membranes, and acetylcholine, which play a role in certain physiological activities such as lipid metabolism, glucose metabolism, signaling through lipid second messengers, enterohepatic circulation of bile and cholesterol, and mitochondrial bioenergetics [78]. Compared with the normal population, T2DM patients have dysfunctions in the metabolism of choline, glucose, lipids, and amino acids. Decreased bioavailability of choline can increase the incidence of NAFLA and can alter glucose metabolism [73]. Decreased serum level of choline in diabetic patients is associated with the gut microbiota [79].

*Firmicutes, Proteobacteria* phyla bacteria and other anaerobic gut microbiota produce enzymes that convert dietary choline into trimethylamine (TMA), which cannot be synthesized by humans. Trimethylamine is an intermediate in the conversion of choline to betaine, methylamine, dimethylamine (DMA), and trimethylamine N-oxide (TMAO), the formation of which is associated with intestinal bacteria action [69, 80]. Flavin monooxygenases 1 and 3 in the liver can metabolize TMA to TMAO, which is associated with impaired renal function, colorectal cancer, diabetes, and cardiovascular disease. Mice fed HFD with 0.2% TMAO had higher fasting insulin level and homeostasis model assessment-estimated insulin resistance and impaired glucose tolerance associated with the hepatic insulin signaling pathway and adipose tissue inflammation [81]. The following bacteria from two phyla (i.e.,* Firmicutes* and* Proteobacteria*) and six genera showed significant choline consumption and TMA accumulation:* Anaerococcus hydrogenalis, Clostridium asparagiforme, Clostridium hathewayi, Clostridium sporogenes, Escherichia fergusonii, Proteus penneri, Providencia rettgeri, *and* Edwardsiella tarda* [77]. Fecal choline connected with* Bacteroides *and negatively with* Prevotella *and* Clostridium*, the secondary BAs deoxycholic acid connected with* Bacteroides*,* Erysipelotrichaceae incertae sedis, *and* Enterococcus*, but negatively with* Prevotella*,* Barnesiella, *and* Alistipes *in mice. Fecal SCFAs connected with* Prevotella*,* Barnesiella, *and* Alistipes *and negatively with* Bacteroides*,* Erysipelotrichaceae incertae sedis *and* Enterococcus *in mice [76].

The leave of trimethylamine* N-*oxide is negatively associated with* Peptococcaceae*,* Prevotella, *and* Faecalibacterium prausnitzii* and is significantly and positively associated with* Allobaculum*,* Candidatus Arthromitus, or Lachnospiraceae* [82]. Reduced levels of* Faecalibacterium prausnitzii* have been associated with obesity, diabetes, and several immune-related diseases [46]. Plasma TMA and TMAO levels are positively correlated with* Clostridiales*,* Ruminococcus*, and* Lachnospiraceae* bacteria in female mice [83].

### 2.4. Amino Acids Metabolism and the Intestinal Flora

Gut bacteria can change bioavailability of amino acids via utilizing certain amino acids originating from alimentary proteins and endogenous proteins and can supply amino acids for a host. Amino acids support growth and survival of gut bacteria and regulate energy and protein homeostasis. Elevated level of amino acids, particularly branched-chain and aromatic amino acids (e.g., phenylalanine and tyrosine), promotes the development of insulin resistance and T2DM.

Certain amino acids, as precursors for the synthesis of SCFAs, are liberated by gut microbiota and are involved in the development of obesity.* Clostridium*,* Bacillus*,* Lactobacillus*,* Streptococcus*, and* Proteobacteria* in the small intestine and* Clostridia*,* Peptostreptococcus* in large intestine can ferment amino acids. This process is associated with protein digestion and subsequently with amino acid absorption. Colonic bacteria primarily use certain amino acids as substrates such as the branched-chain amino acids (BCAAs), lysine, arginine, and glycine. They produce metabolic end-products such as SCFAs and branched-chain fatty acids such as valerate, isobutyrate, and isovalerate. These bacterial metabolites can affect signaling pathways in epithelial cells and in the mucosal immune system, and thereby influence epithelial physiology. The BCAAs, aromatic amino acids (e.g., tyrosine), and other amino acids (e.g., alanine, lysine, and methionine) are positively associated with* Prevotella*,* Alistipes*, and* Barnesiella*, but are negatively associated with* Bacteroides* and* Enterococcus*. In addition,* Prevotellaceae* is significantly enriched in obesity [84].

#### 2.4.1. Branched-Chain Amino Acids and the Intestinal Flora

BCAAs include leucine, isoleucine, and valine. BCAAs are involved in the synthesis of protein, alanine, glutamine, and glucose metabolism and oxidation [85]. Plasma BCAAs are increased in patients with metabolic disorders such as high fasting glucose level and insulin resistance [86]. Isoleucine can increase insulin-independent glucose uptake in vitro and improve glucose metabolism and oxidation. In fasted rats, oral isoleucine reduced the plasma glucose level and increased muscle glucose uptake, which was correlated with decreased messenger ribonucleic acid (mRNA) levels of phosphoenolpyruvate carboxykinase, glucose-6-phosphatase (G6Pase), and G6Pase in isolated hepatocytes [87]. BCAAs are negatively associated with abundance of* Christensenellaceae* and are positively associated with* Blauti*a [46].

#### 2.4.2. Other Amino Acids and the Intestinal Flora

Prediabetic patients reduced levels of amino acids such as l-lysine, l-threonine, betaine, isovaleraldehyde, 2-ketobutyric acid, 2-pyrroloylglycine, and abnormal glutamine, glycine, serine, and threonine [88]. The level of 2-Ketobutyric acid is correlated with glycine, methionine, valine, leucine, serine, threonine and isoleucine, which are associated with diabetes. Some amino acids such as 2-ketobutyric acid, glycine, serine, and threonine have an important role in the TCA cycle [3]. Individuals with obesity or visceral obesity have increased levels of lysine, tryptophan, cystine, and glutamate, and decreased levels of asparagine, citrulline, glutamine, glycine, and serine.

The fasting and 2-hour plasma glucose levels and the homeostasis model assessment of insulin resistance are positively associated with valine, glutamate, and tyrosine levels, but negatively associated with citrulline, glutamine, and glycine levels [89]. The* Photobacterium* genus was negatively associated with alanine, glycine, taurine, whereas* Coprococcus 3* and* Vibrio* genera were positively associated with citrulline [90]. Tryptophan, which is the amino acid precursor for the signaling molecule 5-hydroxytryptophan, is regulated by gut microbiota such as* Bifidobacterium* and* Bacteroides fragilis* [73].* Bacteroides thetaiotaomicron* reduced the plasma glutamate level and improves diet-induced body weight gain and adiposity in mice. This finding suggests that glutamate is negatively associated with* B. thetaiotaomicron* [91].

Some metabolites are also altered in MS. Hippurate, 4-hydroxylphenylacetic acid, and phenylacetylglycine levels are increased and acetate, lactate levels are decreased in urine. These metabolites are associated with obesity and an alteration in gut microbiota [35]. Niacin metabolites such as nicotinuric acid and trigonelline, which are associated with gut microbiota, are connected with obesity and T2DM. Diabetic patients had lower level of trigonelline in association with a change in energy and tryptophan metabolism [69]. Allantoin, an oxidative product of uric acid, was higher in mice with HFD-induced hyperlipidemia, which was associated with oxidative stress [92, 93].

### 2.5. The TCA Cycle and the Intestinal Flora

The intermediates of TCA cycle include citrate, butanedioic acid, citrate, succinate, pyruvate, and cis-aconitate. These intermediates have a vital role in energy metabolism [94]. Pyruvate is an important metabolite in glycolysis and in TCA cycle. It converts pyruvate into acetyl-coenzyme A (acetyl-CoA), which enters TCA cycle. Reduced glycolysis may result in a higher level of glucose. Pyruvate is positively associated with* Coriobacteriaceae* and* Blautia*. Rats with HFD-induced hyperlipidemia increased levels of pyruvate and decreased levels of citrate, succinate, and cis-aconitate and caused dysfunction in glucose metabolism, insulin utilization, and energy metabolism. Increased fatty acid oxidation occurred in rats with hyperlipidemia, which might generate SCFAs or medium-chain fatty acids, and thereby reduced glucose metabolism, and changed energy consumption to lipid oxidation [95]. Decreased levels of pyruvate, citrate, succinate, fumarate, and 2-oxoglutarate and increased levels of pantothenic acid occurred in the serum of HFD-fed mice. In addition, the liver of HFD-fed mice decreased levels of the glycolysis end-products pyruvate and lactate, glutamate and decreased levels of the TCA cycle intermediates citrate, succinate, fumarate, malate, and oxaloacetate [96]. Increased TCA enhances the secretion of GLP-1, which is associated with the depletion of* Firmicutes* and* Bacteroidetes* bacteria [97]. The relationship of gut microbiota and its metabolites are summarized in Table 1.

## 3. The Effect of TCM Treatment on the Intestinal Flora and Its Metabolites in Glycolipid Metabolism Diseases

Experiments have shown that diet control, TCM treatment, and other methods can regulate the intestinal flora structure and its metabolites, reduce inflammatory factors, and relieve insulin resistance, obesity, metabolic disorders [98–100].

### 3.1. TCM Improves SCFAs and the Associated Enrichment of Bacteria

The abundance of SCFAs-producing bacteria in the intestines of patients with glycolipid disorders is decreased. Many Chinese medicines regulate glycolipid metabolism by enriching bacteria producing SCFAs. For example, Rhizoma Coptidis alkaloids regulated the structure of gut microbiota to alleviate hyperglycemia, which might help alleviate inflammation by increasing the level of intestinal SCFAs [101]. Xiexin Tang altered SCFA-producing and anti-inflammatory bacteria such as* Adlercreutzia, Alloprevotella, *and* Barnesiella* in T2DM [102]. Shenling Baizhu powder increased SCFAs-producing bacteria including* Bifidobacterium* and* Anaerostipes* in NAFLD rats [103]. Oral hydroxysafflor yellow A, an active compound from the dried florets of* Carthamus tinctorius* L., increased SCFAs-producing bacteria, including genera* Butyricimonas* and* Alloprevotella* in obesity mice [104]. Huang-Lian-Jie-Du-Decoction enriched SCFAs-producing and anti-inflammatory bacteria, such as* Parabacteroides, Blautia*, and* Akkermansia* in T2DM rats [105]. JinQi Jiangtang tablets enriched* Akkermansia* spp., a bacteria producing SCFAs, and reduced* Desulfovibrio* in T2DM [106]. A water insoluble polysaccharide increased* Lachnospiraceae* and* Clostridium* in ob/ob mice with hepatic steatosis.* Lachnospiraceae* and* Clostridium* can produce butyrate [107]. A specifically designed herbal formula increased* Faecalibacterium* and* Roseburia*, which contained butyrate-producing bacteria. It also enriched SCFA-producing bacteria, including* Gemmiger*, and* Coprococcus* in T2DM [14]. Berberine increased SCFAs in feces and SCFAs-producing microbiota such as* Blautia* and* Allobaculum* in insulin-resistant rats [101]. And Berberine also enhanced SCFAs-producing bacteria such as* Allobaculum*,* Blauti*a,* Bacteroides*,* Butyricicoccus*, and* Phascolarctobacterium* in rats with HFD-induced obesity [36].

### 3.2. TCM Improves BAs and Related Bacteria

TCM ingredients can affect genes expression, and consequently regulate FXR, or structure of intestinal flora to regulate the conversion of BAs in the liver and intestine. Resveratrol significantly increased the abundance of* Lactobacillus* and* Bifidobacterium* and increased the activity of intestinal bile acid hydrolase to promote the catabolism of BAs in the intestine, which promoted the efflux of fecal BAs. The synthesis of BAs in the liver was ultimately promoted [108]. Total saponins in* Gynostemma* have the pharmacological effects of regulating lipid metabolism.* Gynostemma* can regulate BAs metabolic pathways and bile acid layer, and its target may be the nuclear receptor FXR [109]. Pepper extract decreased lipid by regulating BAs in the intestines and liver. It promoted intestinal peristalsis, and increased ileum FXR gene expression, BAs level, and neutral steroid excretion. Meanwhile, it reduced BAs reflux in intestinal circulation, and accelerated cholesterol decomposition by lowering liver FXR expression [83]. Berberine prominently lowered BCAA-producing bacteria, including order* Clostridiales, *families Streptococcaceae*, Clostridiaceae,* and* Prevotellaceae*, and genera* Streptococcus* and* Prevotella* in HFD-fed mice with insulin resistance [110]. Epigallocatechin-3-gallate regulated bile acid signaling and enriched* Akkermansia muciniphila* in diet-increased obesity [111].

### 3.3. TCM Improves TMA/TMAO Levels and Related Bacteria

The compound of vinegar and olive oil (which contains 3, 3-dimethyl-1-butanol) can effectively increase SCFAs levels and block TMAO production in mice [112]. Resveratrol can reduce the level of TMA by remodeling the intestinal flora, thereby affecting lipid metabolism and preventing arteriosclerosis [108].

### 3.4. Effect of TCM on Other Metabolites

Many TCM ingredients act on amino acid metabolism and TCA, and participate in energy metabolism. Turmeric regulated fat production and decomposition pathways to lower blood lipids. Curcumin-treated mice had increased levels of acetate, leucine, isoleucine, valine, alanine, and TCA cycle intermediates (e.g., lemon acid salt, succinate, and cis-aconitate). Berberine is involved in glycolysis and the TCA cycle, amino acid metabolism, vitamin B6 metabolism, and secondary bile acid biosynthesis [113]. Gardenia jasminoides regulated metabolic perturbations in phenylalanine, tyrosine, and tryptophan biosynthesis, tryptophan metabolism, and purine metabolism [114]. Qijian mixture, consisting of Astragalus membranaceus, Ramulus euonymi, Coptis chinensis, and Pueraria lobata, changed related proteins and signaling pathways, including valine, leucine and isoleucine degradation metabolism, aminoacyl-tRNA biosynthesis metabolism and alanine, aspartate, and glutamate metabolism pathways, with significantly enrichment of bacteroidetes [115]. Ferulic acid regulated gut microbiota's composition, including altering the ratio of* Firmicutes* to* Bacteroidetes*, and reduced indole-3-acetic acid level in ApoE-/- mice fed on a high-fat diet [10]. The effect of TCM treatment on intestinal flora and its metabolites are summarized in Table 2.

## 4. Summary

As the number of obese people increases, glycolipid disorders become more common in the future. Glycolipid disorders have a significant impact on the quality of life and the financial resources of the public healthcare system. To date, treatment for T2DM with hyperlipidemia primarily relies on the use of hypoglycemic and lipid-lowering drugs. However, patients are increasingly concerned about potential toxicity and side effects and sometimes the clinical efficacy is unsatisfactory. Intensive studies on complementary and alternative medical therapies have increased the utility of these therapies for treating glycolipid metabolic diseases. Natural medicine, especially Chinese herbal medicine, constitutes a special medical system that is guided by TCM theory and provides effective treatment for glycolipid disorders.

Traditional Chinese herbal medicines are generally administered orally, and their digestion and absorption are inseparable from the intestinal flora. An increasing number of Chinese herbal ingredients have been proven to adjust an individual's intestinal flora, and thereby allow the flora to approximate that of a normal person. This trend is significantly associated with recovery from the diseases. Regulating the intestinal flora is an important way for Chinese medicines to take effect.

Among several metabolites, the SCFAs are most affected by Chinese medicine constituents such as berberine, Ganoderma lucidum, and resveratrol, which enrich SCFAs-producing bacteria, and increase intestinal SCFAs. The production of SCFAs can relieve insulin resistance and alleviate disorders of glycolipid metabolism. In addition, several traditional Chinese medicines can regulate BAs metabolism, upregulate FXR levels, increase insulin sensitivity, inhibit fat synthesis, and reduce serum triglyceride and cholesterol levels. These actions are closely associated with the regulation of the related gut microbiota. The level of TMA/TMAO is correlated with glycolipid metabolism and is an important marker and potential therapeutic target for cardiovascular disease. By intervening intestinal flora and its metabolites, TCM can achieve hypoglycemic and lipid-lowering effects and effectively reduce the incidence of cardiovascular events. In addition, TCM monomers are also widely involved in intestinal amino acid metabolism and in the TCA cycle.

In summary, TCM can regulate the body's glycolipid metabolism through the intestinal flora and its metabolites, and can achieve a therapeutic effect in people with glycolipid metabolic disorders. A Chinese medicine practitioner prescribes medicine, based on a patient's symptoms such as abdominal feeling, body shape, and stool shape. For patients with polydipsia, obesity, diabetes, and hyperlipidemia, TCM often eliminates digestion and promotes gastrointestinal motility, while relieving symptoms. TCM has a vital role in reducing sugar and fat levels. It can regulate glycolipid metabolism by altering the intestinal flora and its metabolites.

This review still has some limitations. Some conclusions are based on clinical trials with low levels of evidence, which causes biases. In addition, many studies lack evidence on the intestinal flora and its metabolites. The mechanism of TCM in treating glycolipid metabolic disorders needs further research. In the future, carefully designed, large-scale, high-quality randomized controlled trials are needed to evaluate the effects of TCM on glycolipid metabolism. In addition, in-depth studies need to be conducted from the perspective of the flora to detect relevant targets in metabolic pathway and to clarify the biological mechanisms of Chinese medicine. Ideas need to be provided for preventing and treating glycolipid metabolism diseases.

## Figures and Tables

**Table 1:** Metabolites of gut microbiota.

Metabolites	Related gut microbiota	Effects
Fatty acids	Phyla: *Firmicutes,* *Bacteroidetes,* *Actinobacteria,* *Proteobacteria,* *Tenericutes,* *Verrucomicrobia* Class: *Clostridia* Order: *Clostridiales* Family: *Christensenellaceae,* *Lachnospiraceae,* *Ruminococcaceae,* *Negativicutes,* *Enterobacter hormaechei* Genus: *Akkermansia,* *Lactobacillus,* *Bacteroides,* *Bifidobacterium,* *Propionibacterium,* *Eubacterium,* *Lactobacillus,* *Clostridium,* *Roseburia,* *Lachnospira,* *Prevotella,* *Butyricicoccus,* *Blautia,* *Phascolarctobacterium,* *Butyrivibrio,* *Faecalibacterium prausnitzii*, *Streptococcus* Species: *Haemophilus parainfluenzae*, *Coprococcus,* *Peptococcaceae*	Provide energy to maintain intestinal epithelial cell function, regulate glucose homeostasis, lipid metabolism, and insulin sensitivity.

BAs	Phyla: *Firmicutes,* *Clostridium*, *Bacteroides*, *Lactobacillus*, *Bifidobacterium*, *Enterococcus* Genus: *Listeria,* *Escherichia,* *Eubacterium,* *Peptostreptococcus,* *Ruminococcus,* *Fusobacterium,* *Peptococcus,* *Pseudomonas,* *Blautia,* *Coprococcus,* *Intestinimonas,* *Lactococcus,* *Roseburia* Others: *Eggerthella*	Regulate lipid homeostasis, glucose utilization, energy, drug metabolism, inflammation, detoxification, and immunity.

Choline	Phyla: *Firmicutes,* *Proteobacteria,* *Bacteroides,* *Prevotella,* *Clostridium* Order: *Clostridiales* Genus: *Anaerococcus hydrogenalis,* *Clostridium asparagiforme,* *Clostridium hathewayi, * *Clostridium sporogenes,* *Escherichia fergusonii*, *Proteus penneri*, *Providencia rettgeri,* *Edwardsiella tarda,* *Erysipelotrichaceae incertae sedis,* *Enterococcus,* *Prevotella,* *Barnesiella,* *Alistipes,* *Peptococcaceae,* *Allobaculum,* *Candidatus Arthromitus,* *Ruminococcus* Family: *Faecalibacterium prausnitzii,* *Lachnospiraceae*	Ingredient of cell membranes, mitochondrial membranes, and acetylcholine; play a role in certain physiological activities such as lipid metabolism, glucose metabolism.

Amino Acids	Phyla: *Proteobacteria * Class: *Clostridia* Family: *Prevotellaceae,* *Christensenellaceae* Genus: *Bacteroides,* *Clostridium,* *Bacillus,* *Lactobacillus,* *Streptococcus,* *Peptostreptococcus,* *Prevotella,* *Alistipes,* *Barnesiella,* *Enterococcus,* *Blautia*, *Photobacterium,* *Coprococcus, Vibrio,* *Bifidobacterium,* *Bacteroidesfragilis,* *Bacteroides thetaiotaomicron*	Support growth and survival of gut bacteria; regulate energy and protein homeostasis; involved in the synthesis of protein, alanine, glutamine, and glucose metabolism and oxidation.

TCA	Phyla: *Firmicutes,* *Bacteroidetes* Family: *Coriobacteriaceae* Genus: *Blautia*	Have a role in energy metabolism, glucose metabolism, insulin utilization.

**Table 2:** The effect of TCM treatment on intestinal flora and its metabolites.

TCM treatment	Effect on intestinal flora	Effect on metabolites
Xiexin Tang	Altered* Adlercreutzia, * *Alloprevotella, Barnesiella*	Altered SCFAs
Shenling Baizhu powder	Increased* Bifidobacterium,* *Anaerostipes*	Increased SCFAs
hydroxysafflor yellow A	Increased* Butyricimonas,* *Alloprevotella*	Increased SCFAs
Huang-Lian-Jie-Du-Decoction	Increased* Parabacteroides, * *Blautia,* *Akkermansia*	Increased SCFAs
JinQi Jiangtang tablets	Increased *Akkermansia*, decreased *Desulfovibrio*	Increased SCFAs
water insoluble polysaccharide	Increased *Lachnospiraceae, Clostridium*	Increased butyrate
a specifically designed herbal formula	Increased *Faecalibacterium, Roseburia* Increased *Gemmiger, Coprococcus*	Increased butyrate Increased SCFAs
Berberine	Increased *Blautia, Allobaculum, Bacteroides, Butyricicoccus, Phascolarctobacterium*	Increased SCFAs
	Decreased *Clostridiales, Streptococcaceae, Clostridiaceae, Prevotellaceae, Streptococcus, Prevotella*	Decreased BCAA
Resveratrol	Increased *Lactobacillus, Bifidobacterium, *	Increased BAs
Epigallocatechin-3-gallate	Increased *Akkermansia muciniphila*	Regulated bile acid signaling
Ferulic acid	Altered the ratio of *Firmicutes* to *Bacteroidetes*	Reduced indole-3-acetic acid
